# Multiscale heterogeneous optimal lockdown control for COVID-19 using geographic information

**DOI:** 10.1038/s41598-022-07692-5

**Published:** 2022-03-10

**Authors:** Cyrus Neary, Murat Cubuktepe, Niklas Lauffer, Xueting Jin, Alexander J. Phillips, Zhe Xu, Daoqin Tong, Ufuk Topcu

**Affiliations:** 1 The University of Texas at Austin, Austin, TX USA; 2 The University of California, Berkeley, Berkeley, CA USA; 3 Arizona State University, Tempe, AZ USA

**Keywords:** Applied mathematics, Computational science, Control theory, Dynamic networks, Epidemiology

## Abstract

We study the problem of synthesizing *lockdown policies*—schedules of maximum capacities for different types of activity sites—to minimize the number of deceased individuals due to a pandemic within a given metropolitan statistical area (MSA) while controlling the severity of the imposed lockdown. To synthesize and evaluate lockdown policies, we develop a multiscale susceptible, infected, recovered, and deceased model that partitions a given MSA into geographic subregions, and that incorporates data on the behaviors of the populations of these subregions. This modeling approach allows for the analysis of heterogeneous lockdown policies that vary across the different types of activity sites within each subregion of the MSA. We formulate the synthesis of optimal lockdown policies as a nonconvex optimization problem and we develop an iterative algorithm that addresses this nonconvexity through sequential convex programming. We empirically demonstrate the effectiveness of the developed approach by applying it to six of the largest MSAs in the United States. The developed heterogeneous lockdown policies not only reduce the number of deceased individuals by up to 45 percent over a 100 day period in comparison with three baseline lockdown policies that are less heterogeneous, but they also impose lockdowns that are less severe.

## Introduction

**Figure 1:**
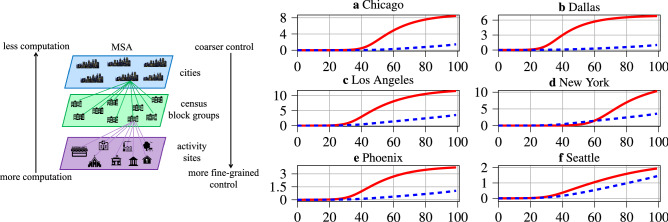
Left: Visualization of the multiple scales of pandemic control strategy synthesis. Right: Resulting SIRD dynamics for the considered MSAs. The y-axes denote the cumulative number of infected people in millions, and x-axes denote the time in days. The blue and red curves visualize the cumulative number of infected people with and without an optimal lockdown policy, respectively.

The COVID-19 pandemic has caused over 180 million confirmed cases and over 3.96 million deaths globally as of June 30, 2021. Since the outbreak of COVID-19, various public health control strategies have been proposed and tested against the coronavirus SARS-CoV-2. However, existing COVID-19 control synthesis approaches typically either apply optimal control techniques using models that largely neglect interactions between individuals living in different geographic regions, or focus on evaluating relatively simple control strategies with no optimality guarantees.

Approaches that apply optimal control techniques largely rely on models that neglect important spatial-temporal dynamics associated with regional differences in the pandemic’s spread, and with people’s movement^1–9^. However, studies of the spread of COVID-19 in India, France, and Italy have demonstrated the important role that heterogeneity between geographic regions plays in attempts to predict and to control the pandemic^10–12^. It is essential that any interventions to slow the spread of the COVID-19 distinguish between different geographic regions, which may have differing demographics, available medical equipment, and rates of transmission. The exclusion of such geographic information in the modeling and analysis of control policies may result in significant discrepancies between the model-predicted outcomes, and the practical results observed when the control policies are deployed.

Conversely, the research that incorporates detailed spatial-temporal geographic information does not focus on the synthesis of optimal control policies^13–16^. Instead, these works evaluate given control policies without any consideration of their optimality. Note that such optimality becomes very important when our objective is not only to minimize deaths, but also to reduce the severity of the imposed lockdown.

In this work, we study the synthesis of optimal *lockdown policies* that explicitly take such geographic consideration into account. A lockdown policy specifies the maximum number of allowable concurrent visitors to various types of *activity sites* accross a given metropolitan statistical area (MSA). We define activity sites to be the physical locations throughout the MSA, such as grocery stores and fitness centers, where interactions between members of the population frequently occur and thus where the disease is likely to spread.

**Figure 2:**
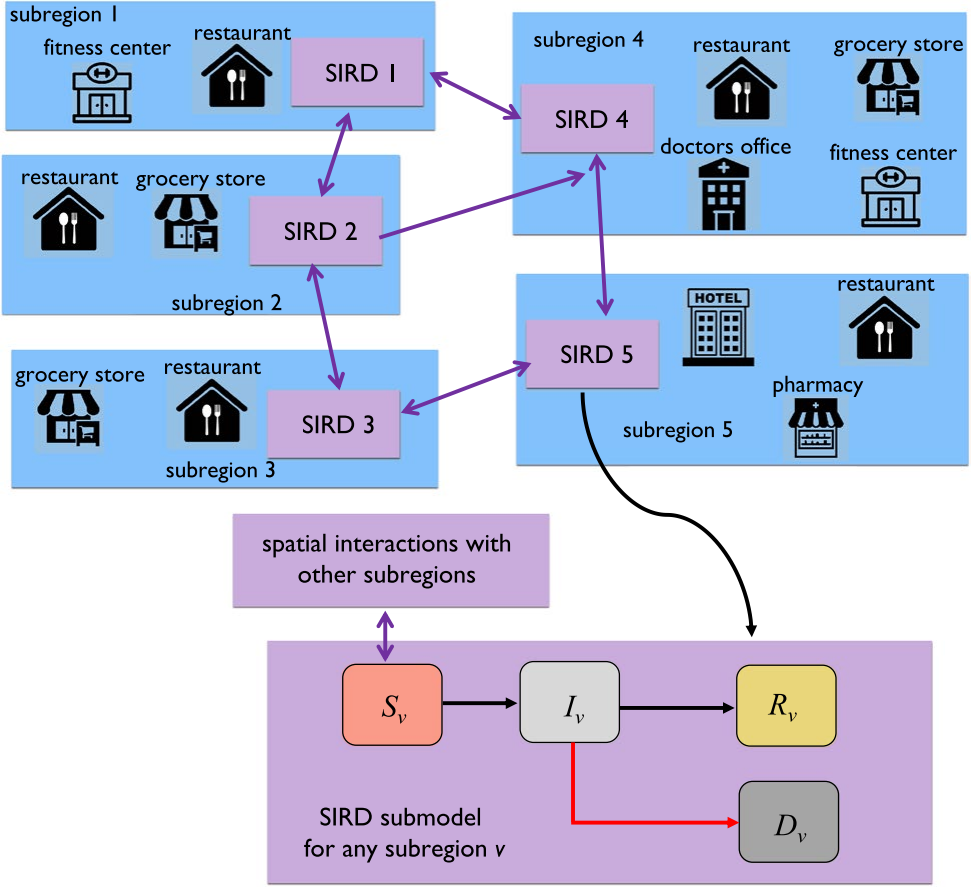
The multiscale SIRD model with spatial interactions among different subregions (e.g., cities).

To synthesize optimal lockdown policies while incorporating the aforementioned geographic considerations, we first develop a multiscale susceptible, infected, recovered, and deceased (multiscale SIRD) model of the spread of a disease through a given MSA. The model explicitly incorporates geographic data describing the spatial distribution of the population, and interactions between individuals from different subregions within the MSA. The visualization on the left of Fig. 1 illustrates the geographic and multiscale nature of the modeling approach; each MSA is partitioned into subregions, and each subregion contains a collection of different types of activity sites. The spread of the disease through each individual subregion is captured by a separate SIRD model. All potential inter-regional spread of the disease—driven by the frequency at which members of the distinct subregion populations come into contact—is then modeled by interaction terms between the SIRD submodels, illustrated by the arrows in Fig. 2.

By explicitly modeling the activity sites at which the disease is likely to spread, the developed approach allows for the synthesis of optimal *heterogeneous* lockdown policies. Each of these policies specifies the allowable number of visitors to a specific type of activity site within a given subregion. We formulate the problem of computing optimal heterogeneous lockdown policies as a nonconvex optimization problem. We then develop an iterative algorithm addressing this nonconvexity through sequential convex programming^17^. Building on related results^17–20^, we linearize the underlying nonconvex problem around the solution from the previous iteration and check whether the synthesized policy obtains a better objective value. The algorithm can synthesize a heterogeneous lockdown policy that is locally optimal with respect to the nonconvex optimization problem.

We evaluate the developed modeling framework and optimal policy synthesis algorithm using data from six of the largest MSAs in the United States: Phoenix, New York, Chicago, Los Angeles, Dallas-Fort Worth, and Seattle. Figure 1 visualizes the difference in the cumulative number of infections within each MSA’s population that results from the application of optimal lockdown policies, in comparison with imposing no lockdown. The experimental results, discussed further below, demonstrate that the developed optimal heterogeneous lockdown policies not only result in far fewer deaths in each MSA, but also in lockdown policies that are much less severe than their homogeneous counterparts, which impose the same lockdown to all types of activity sites and to all subregions.

## Related work

### COVID-19 epidemic modeling

Since the initial outbreak of COVID-19, there has been extensive research into modeling its spread within a population^21–26^. Commonly used *compartmental models* partition the population into labeled groups, each of which describes a different phase of infection^2,27–32^. For example, the SIR model separates the population into three compartments, those who are susceptible to the virus $(S)$, those who are currently infectious $(I)$, and those who have been removed from the model’s consideration $(R)$^33^. Given such a partition of the population, systems of ordinary differential equations are often used to model the dynamics of the disease’s spread. By including additional compartments in the model, and thus refining the partition of the population into more detailed categories, predictions and analysis of specific changes and quantities of interest can be made. For example, partitioning the population into different age categories allows for the analysis of age-specific targeted lockdown policies^2^. Giordano et al.^32^ consider eight categories in their compartmental model, allowing for discrimination between infected individuals depending on whether they have been diagnosed and on the severity of their symptoms; this refinement aims to enable the model to reflect the observed high number of asymptomatic individuals who are still able to cause transmissions.

While such compartmental models provide an easy-to-interpret means of analyzing the spread of COVID-19, they may only be used in the context of relatively large populations. Conversely, agent-based models instead encode rules for agents—simulated members of the population—to follow. These models simulate the spread of the disease resulting from these behaviors^34–40^. Agent-based models allow for simulation of the effectiveness of behavioral interventions on the level of individual members of the population, such as mask-wearing and social distancing requirements within an enclosed space.

Several papers have considered further extensions of compartmental models. Chang et al.^41^ model transmission within a network where households make contact at common *points of interest*. Karaivanov^42^ incorporates a social network model with an SIR model to provide a more realistic model of the interactions within a population, as opposed to the uniform mixing assumed by most SIR models. However, none of the above papers consider the problem of optimal control. Table 1 summarizes several representative references for COVID-19 modeling.

**Table 1:** List of representative references for COVID-19 modeling.

Compartmental	Agent-based	Network-based	Other
SIR^2^, SEIR^27^, SIQS^30^, SUQC^31^, SIDARTHE^32^	^34–40^	^12,41–44^	^45–50^

**Table 2:** List of representative references for COVID-19 control.

Control	Single-scale	Optimality	Geographic information	Demographic Information
Lockdown	^1,2,13,14,51^	^1,2^	^13,14^	^2,51^
Testing	^3,15,52^	^3^	^15^	^52^
Vaccination	^4–9,16^	^4–6^	^16^	^4–6^

### COVID-19 related control

Several papers have investigated the problem of control analysis for various COVID-19 related policies, including lockdowns, testing, and vaccine distribution. Sardar et al.^53^ use compartment-style pandemic models to study the effect of lockdowns on the spread of COVID-19. However, they do not synthesize lockdown control policies, nor do they study geographically heterogeneous lockdowns. Chatzimanolakis et al.^54^ and Buhat et al.^55^ both study the problem of optimally distributing test kits under limited supply. Other works study the trade-offs between focusing allocation of vaccinations to either high-risk or high-transmission age-groups in the context of SIR models^3–6^. Goldenbogen et al.^56^ study a human-human interaction network and analyzes the optimal policy for vaccine distribution.

Similar to our work, several papers have studied the problem of synthesizing or evaluating lockdown policies within various epidemiological models to balance the tradeoff between viral spread and economic impact. Alvarez et al.^1^ study the problem of minimizing the deceased people in a basic SIR model while controlling the impact on the economy. Acemoglu et al.^2^ extends this work to consider differing dynamics and control among age groups. Both of these works, along with other COVID-19 control-related papers^13,14,41,51,57,58^, only consider a spatially homogeneous population and control policy. Similar to the regional model considered by Della Rossa^12^, we consider a hierarchical model allowing for region-specific dynamics and control. Table 2 summarizes several representative references for COVID-19 control.

## Methods

We formulate a multiscale SIRD pandemic model that uses a directed graph to capture the spread of the disease through a given MSA. The model also captures how the disease’s spread is affected by the lockdown of various types of activity sites. Each node of this graph represents a distinct geographic subregion and contains an independent SIRD model, which captures the spread of COVID-19 within the corresponding population. We represent the interactions between the populations of the subregions, i.e., the SIRD models, by the directed edges in the graph. The weight of a given edge encodes the fraction of the origin subregion’s population that frequently interact within the subregion represented by the destination node; these weights are obtained from SafeGraph data^59^.

In this work, we assume that all interactions causing the spread of COVID-19 within the MSA population occur at various types of activity sites. These activity sites represent the physical spaces within a community in which it is common for people, often from different households and potentially from different subregions, to gather in close proximity. In this work, similarly to Giordano et al.^32^, we include the following types of activity sites in our model: *Grocery stores*, *restaurants*, *fitness centers*, *hotels*, *pharmacies*, and *physicians*.

**Figure 3:**
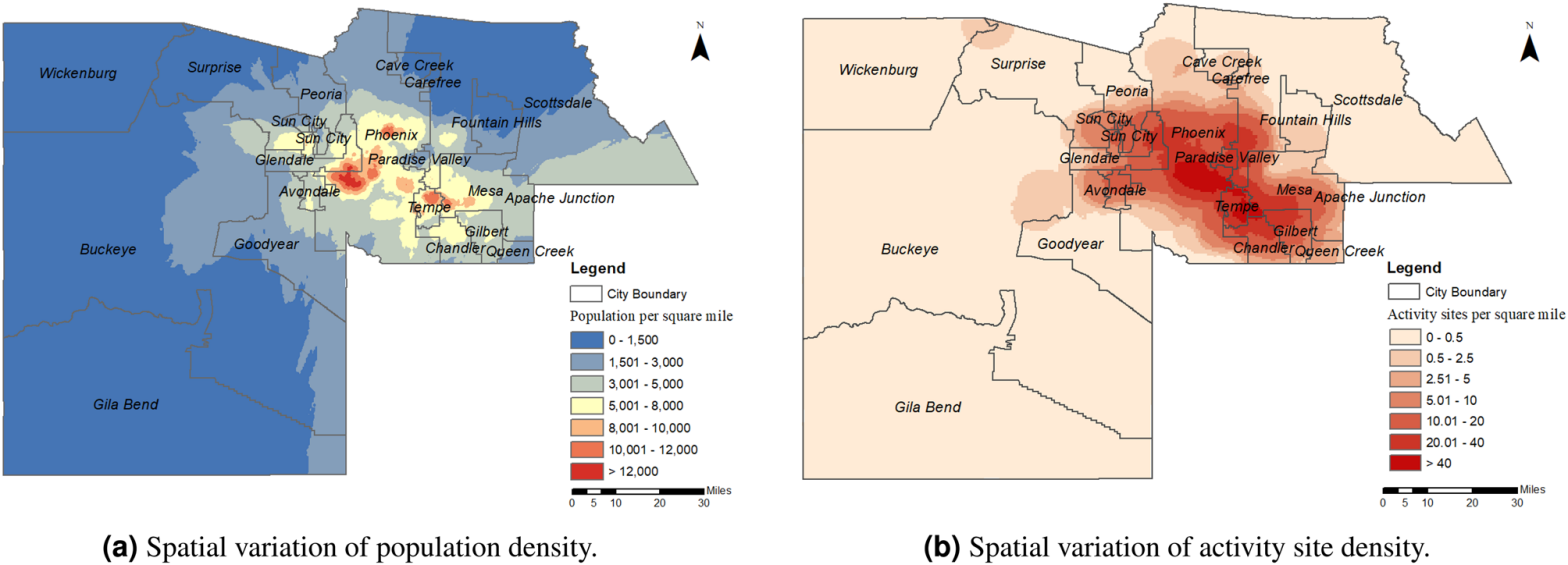
Spatial variation of the population and activity site densities in the Pheonix MSA. The developed multiscale SIRD pandemic model partitions the MSA into subregions according to the visualized subregion boundaries.

We used the foot traffic information of activity sites in April 2020, collected from SafeGraph, to estimate the *demand rates* for each type of activity site within each subregion. The demand rates are defined as the average number of people from a given subregion that visit a particular type of activity site per day. For each type of activity site within each subregion, we then use the corresponding estimated demand rate to define a *nominal capacity*, which represents the maximum number of visits per day that can be sustained by all activity sites of this type within the particular subregion. To help visualize the how the multiscale SIRD model partitions an example MSA into subregions, Fig. 3 illustrates the boundaries used to define the subregions of the Phoenix MSA, as well as the spatial variation of the population and activity site densities, obtained from SafeGraph data.

To control the spread of the disease we specify a separate time-varying lockdown policy for each type of activity site within each subregion. At each point in time, the lockdown policy takes a value between 0 and 1, representing a fraction of the nominal capacity of the corresponding activity site type and subregion. For example, a lockdown policy might specify that all grocery stores within a particular subregion of the MSA may only allow half as many visitors per day as their nominal capacity. On the other hand, in a neighboring subregion, restaurants may only serve up to a quarter of their nominal capacity. So, specifying the lockdown for activity sites reduces their allowable number of visitors per day. This in turn reduces the spread of the disease in the model by reducing the number of interactions occurring in the population. However, in response to the reduction in available capacity at the various activity sites, we assume that members of the population will begin to travel to other subregions. Specifying lockdown policies may therefore also indirectly influence the inter-regional interactions occurring throughout the MSA.

We assume that the selected lockdown policy cannot blindly shut down all activity sites. In particular, we enforce the constraint that, after accounting for the travel between subregions, there must be enough capacity throughout the MSA to satisfy all of the existing demand. This constraint corresponds to the idea of imposing a lockdown while still ensuring that the needs of the population are being met. For example, every member of the population should have the chance to shop for groceries.

Our objective is to solve for a heterogeneous lockdown policy that minimizes the number of deaths in the MSA over some finite time horizon, while limiting the severity of the imposed lockdown. We formulate the computation of an optimal lockdown policy as a nonlinear optimization problem. The variables of the optimization problem encode, at each moment in time, the number of people in each subregion that are susceptible, infected, recovered, and deceased, the fraction of each subregion’s population that visit activity sites within each of the subregions, and the lockdown value for each type of activity site within each subregion. The objective of the optimization problem is a weighted sum of the cumulative deaths in the MSA and the economic cost of the lockdown policy over a finite time horizon. We model the economic cost of imposing a lockdown on a given type of activity site within a given subregion as being proportional to the frequency at which it is visited by members of the MSA’s population. We represent the relative weight of the economic cost in the optimization objective by an *economic impact parameter*. A higher value of this parameter implies a higher relative weight on the economic cost in the objective.

The resulting optimization problem is nonconvex due to the nonlinear dynamics of the multiscale SIRD model. In general, it is computationally hard to compute an optimal lockdown policy for such nonconvex problems^19^. To compute a locally optimal solution, we successively *linearize* the nonconvex optimization problem by computing a local approximation of the problem at each iteration^17–20^. We provide more details on the formulation of the multiscale pandemic model, on the resulting optimization problem, and on our solution approach in the supplementary material.

## Results

In this section, we implement the developed approach using SafeGraph data from Phoenix, Seattle, New York, Chicago, Los Angeles, and Dallas-Fort Worth MSAs. We show detailed results for the Phoenix MSA in this section, and we include the results for the other MSAs in the supplementary material.

**Figure 4:**
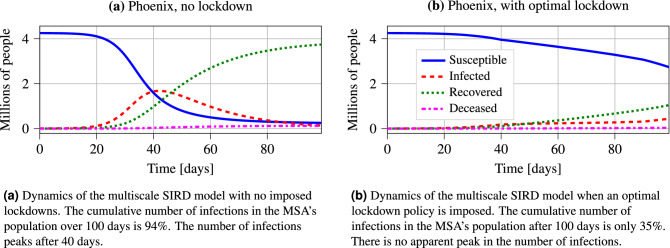
Comparison of the dynamics of the multiscale SIRD model for the Phoenix MSA when no lockdown policy is imposed, and when a computed optimal lockdown policy is imposed. We note that the plots illustrate the current number of susceptible, infected, recovered, and deceased members of the MSA population, as opposed to the cumulative sums of these values.

**Figure 5:**
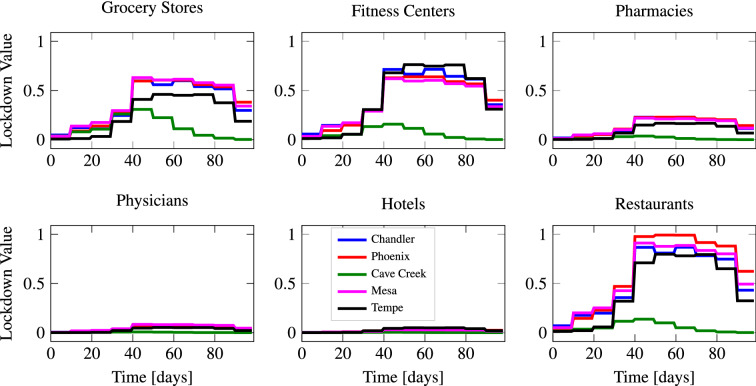
Visualization of the lockdown policies for different types of activity sites and for five representative subregions in the Phoenix MSA. Higher values correspond to lockdowns that are more restrictive. The populations of the five representative subregions, listed in decreasing order, are: Phoenix ($1.67 \times 10^{6}$), Mesa ($5.46 \times 10^{5}$), Chandler ($2.67 \times 10^{5}$), Tempe ($1.82 \times 10^{5}$), and Cave Creek ($6.20 \times 10^{3}$). For all activity sites, we observe that the average lockdown value is higher for subregions with larger populations. We also note that the lockdown value is significantly higher for certain activity sites due to their large demand rates.

In Figs. 4a,b, we show the dynamics of the susceptible, infected, recovered, and deceased people within the Phoenix MSA over a time horizon of 100 days. We observe that without any lockdown, the cumulative number of infected people in the Phoenix MSA by the end of the time horizon is $94\%$ of the total population. Furthermore, the instantaneous number of infected individuals peaks at around 1.7 million after 40 days, which is roughly $40\%$ of the MSA’s total population. Conversely, after imposing an optimal heterogeneous lockdown policy, we observe that the peak number of instantaneous infections is significantly lower than in the case without lockdown. Under an optimal lockdown policy, the instantaneous number of infected individuals plateaus at 0.3 million after 40 days instead of peaking at 1.7 million. As well as flattening the peak number of instantaneous infections, we observe that the imposed lockdown significantly reduces the cumulative number of infected individuals over the time horizon. The cumulative number of infections when an optimal lockdown is imposed is only 35% of the infections when no lockdown is imposed.

**Figure 6:**
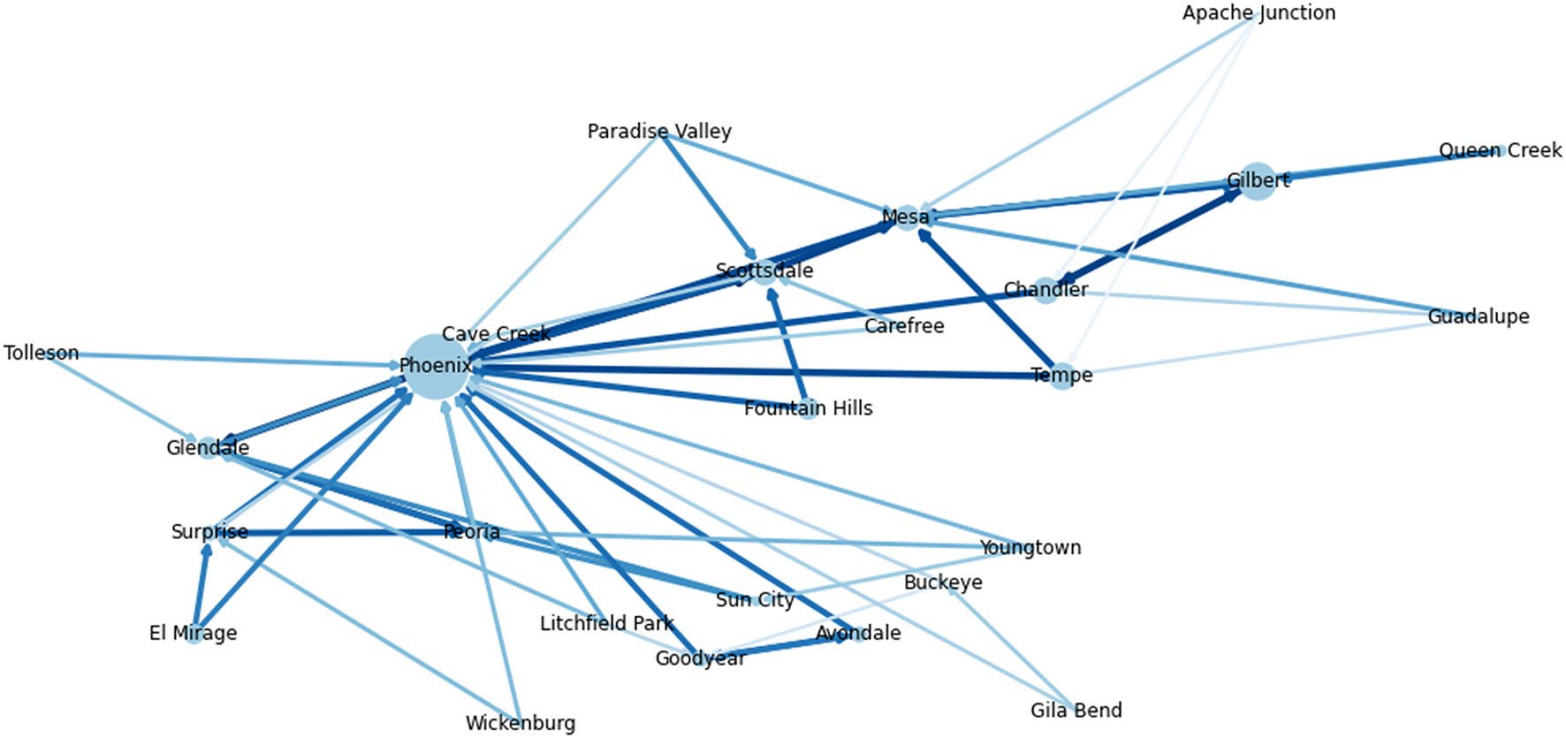
Visualization of the average number of interactions between subregions that results from the solution to the optimization problem for Phoenix MSA. Each node in the visualization graph represents a subregion; node sizes are proportional to the population of the corresponding subregion. Directed edges between subregions visualizes interactions between their populations. The direction of the edge represents the direction of travel: members of the origin subregion’s population visit activity sites within the target subregion, and then return to their home subregion. Increasing edge widths and darker colors for the edges represent a larger number of people that are traveling between subregions.

For each of the six types of activity sites included in the multiscale SIRD model, Fig. 5 illustrates the imposed optimal lockdown policies for five representative subregions within the Phoenix MSA. For all types of activity sites, we observe that the imposed lockdown values peak between 40 and 60 days, before gradually decreasing towards the end of the time horizon. We note that this peak in the lockdown policies coincides with the peak number of infections that occurs when no lockdowns are imposed. The imposed lockdowns in Cave Creek, the subregion with the smallest population in the Phoenix MSA, are much less severe than the lockdowns in the other subregions. Finally, we observe that the lockdowns imposed on grocery stores, fitness centers, and restaurants are much more severe than those imposed on pharmacies, physicians, and hotels. The former three types of activity sites all have much higher demand rates, obtained from SafeGraph data, than the latter three. This pattern indicates a relationship between the frequency at which each type of activity site is visited, and the severity of optimal lockdown policies. This particular relationship is explored further below.

Figure 6, we visualize the average number of interactions between the populations of the different subregions that results from the solution to the optimization problem. Each directed edge represents people from the origin subregion visiting activity sites in the target subregion. The width and color of the edges visualize the average number of such visits. The size of each node is proportional to the size of the population of the corresponding subregion. We note that the edges in Fig. 6 illustrate frequent interactions between the populations of different subregions, not permanent travel that changes the sizes of the populations. The types of activity sites we consider are generally *daily* activities; we assume that after visiting an activity site in another subregion, members of the population will return to their home subregion.

In the remainder of this section we examine the benefits of heterogeneous lockdown policies, the tradeoff between the number of infected individuals and the economic cost of the imposed lockdowns, and the tradeoff between the accuracy of the multiscale SIRD model and the computational performance of the optimization algorithm. For consistency, we present these results only for the Phoenix MSA.

**Figure 7:**
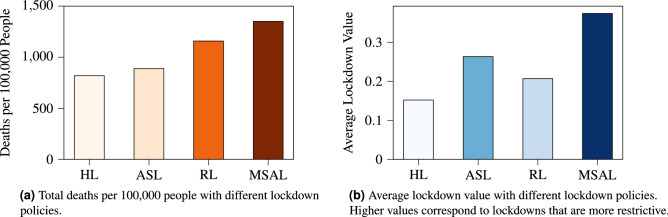
Comparison of the number of deaths and average lockdown values resulting from different classes of lockdown policies. Utilizing the HL policies results in the smallest average lockdown value while also inducing the least number of cumulative deaths.

### Illustrating the benefits of heterogeneous lockdown policies

In Figure 7, we compare the results of four different classes of lockdown policies, each of which is computed using the optimization algorithm described in the Methods section and in the supplementary material, however, with various additional constraints. More specifically, we consider the four different classes of lockdown policy detailed below. All of the considered lockdown policies may specify different lockdown values for different points in time.

- Heterogeneous lockdown (HL): Separate lockdowns are imposed for each type of activity site within each subregion.
- Regional lockdown (RL): Each subregion may impose different lockdowns, but within a given subregion, all activity sites impose the same lockdown.
- Activity site lockdown (ASL): Each type of activity site may impose different lockdowns, but the lockdown for a particular type of activity site is identical across all of the subregions.
- MSA lockdown (MSAL): All activity sites within all subregions impose the same lockdown.

Figure 7a, we note that the HL policies result in fewer deaths compared to the ASL, RL, and MSAL policies, all of which are less heterogeneous than HL. For instance, the MSAL policies result in 65% more deaths than the HL policies over the course of the $100$ day time horizon. We also observe from Fig. 7b that the HL policies incur the lowest average lockdown values, i.e. the lowest lockdown values per time step, averaged over all activity sites and subregions. By allowing the lockdown policies to vary heterogeneously over the different activity sites within the different subregions, we not only reduce the number of deceased indiviuals, but also impose lockdowns that are, on average, less severe than the RL, ASL, and MSAL policies.

Practically speaking, the reason that HL policies are more performant than their more homogeneous counterparts, is because they allow for more granular control of the lockdowns imposed throughout the MSA. This granularity can help to individually address different geographic, demographic, and epidemiological characteristics of the disease’s spread across the different subregions and types of activity sites. Mathematically, this intuitive explanation corresponds to the fact that the class of all HL policies is a superset of the classes of all ASL, RL, and MSAL policies, respectively. That is, any ASL, RL, or MSAL policy can also be considered to be an HL policy. For this reason, it must be the case that the optimal HL policy is at least as performant as the optimal policy from each of these other classes.

### Examining the tradeoff between the number of infected individuals and the economic cost of the imposed lockdowns

**Figure 8:**
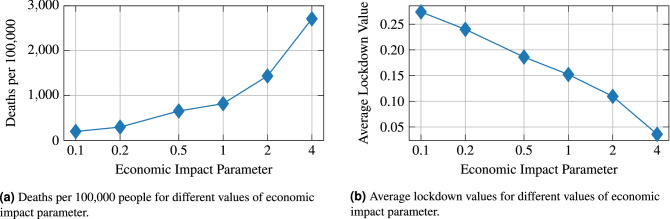
Comparisons of the results of using different values for the economic impact parameter when solving for optimal lockdown policies.

Figure 8, we compare the results of optimal policies that arise from different values of the economic impact parameter, described in the Methods section. We observe that as the value of the economic impact parameter increases, assigning more weight to the economic cost of the imposed lockdowns in the optimization objective, the resultling average lockdown value decreases. Practically speaking, a lower average lockdown value corresponds to lockdown policies that are less restrictive—i.e. the allowed capacities of the activity sites throughout the MSA remain higher. While such policies may be desirable from an economic standpoint, they also result in more interactions between members of the MSA’s population. As demonstrated in Fig. 8a, this leads to a higher cumulative number of deaths by the end of the considered time horizon. So, a clear tradeoff exists between the severity of the disease’s spread throughout the population, and the cost of the imposed lockdown policies.

### Effects of different number of outgoing edges for each subregion

In order to improve the computational performance of the optimization algorithm, we may limit the number of *outgoing edges* from each subregion in the multiscale SIRD model of the MSA. An outgoing edge from subregion $i$ to subregion $j$ in the model implies that people in subregion $i$ can travel to subregion $j$ to visit an activity site. By pruning edges from the model, the number of variables in the optimization problem can be significantly decreased, at the expense of a less accurate representation of the disease’s inter-regional spread. In Table 3, we compare the results of the optimal policies obtained when the multiscale SIRD model is restricted to only include 3, 4, or 5 outgoing edges from each subregion. For a detailed description of how we obtain the outgoing edges to keep in the model, see the supplementary material.

We observe that by including 5 outgoing edges per subregion in the model instead of 3, we can solve for an optimal lockdown policy with 26% fewer deaths. Conversely, the average lockdown value is very similar for the different number of edges. In other words, including more outgoing edges per subregion leads to solutions that reduce the number of deaths, without imposing more severe lockdown policies. However, this benefit comes at the expense of an increase in computation time; the computation times for 4 and 5 edges are 80% and 240% larger than for 3 edges, respectively.

We also note that the lockdown value and the number of deaths are very similar for the models that use 4 and 5 outgoing edges for each subregion. This is likely because our methods compute a locally optimal solution as opposed to a globally optimal one, which would be intractable.

### Relationship between average lockdown value and the demand rate for types of activity sites

**Figure 9:**
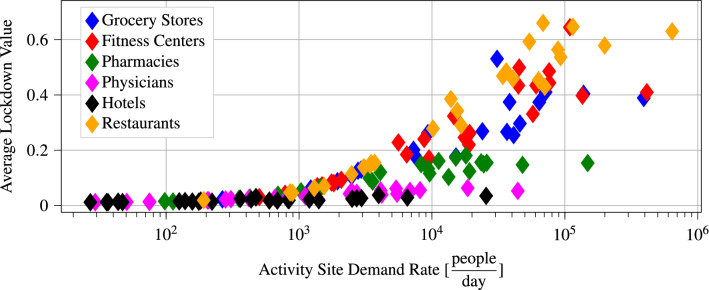
Average lockdown value as a function of the demand rate of different activity sites. Each point in the plot shows the time average of the optimal lockdown policy for a particular type of activity site within a particular subregion. We note that the demand rates are plotted in log scale. We observe a clear increasing relationship between the optimal average lockdown value and the demand rate of a given activity site.

Figure 9 plots the demand rate for each type of activity site within each subregion, as well as the average value of the corresponding optimal lockdown policy. We observe an increasing relationship between these two quantities which indicates that, in general, the solution to the optimization problem described in the Methods section results in more severe lockdown policies for activity sites that are visited more frequently. Intuitively, this results in a redirection of people away from crowded activity sites towards less busy ones, reducing the potential interactions between members of the population.

**Table 3:** Number of deaths and average lockdown value resulting from a different number of outgoing edges per subregion in the multiscale SIRD model of the Phoenix MSA. By using a model incorporating more edges, we can reduce the deaths by up to 26%, without inducing a significant increase in the average lockdown value.

	3 edges	4 edges	5 edges
Deaths per 100,000	819	607	620
Average lockdown value	0.15	0.19	0.19
Number of edges in adjacency matrix	84	109	132

## Discussion

We present a modeling framework and an optimization algorithm for the synthesis of multiscale heterogeneous lockdown policies. Our numerical results demonstrate the effectiveness of these lockdown policies, which individually address the different geographic, demographic, and epidemiological characteristics of the disease’s spread across the different subregions and activity sites within an MSA. The resulting policies provide practical and actionable insights surrounding the incorporation of geographic information into epidemic control strategy synthesis.

Through our numerical results, we demonstrate that lockdown policies that distinguish between different activity sites and geographical subregions not only reduce the number of deceased individuals due to the pandemic but also decrease the severity of the induced lockdowns across the entire MSA, in comparison to less heterogeneous lockdown policies. This result highlights the importance of heterogeneous control strategies in slowing the spread of the pandemic. We also observe an increasing relationship between the demand rate of a particular type of activity site, and the average lockdown value that is assigned to those activity sites by the optimal heterogeneous lockdown policy. Similarly, we observe that the activity sites within subregions with large populations are locked down more severely than those in subregions with small populations. Finally, we observe that the optimal lockdown policy tends to become most severe at the point in time when the number of infections in the population is at its peak value, before gradually easing up over time. In summary, our numerical results suggest that lockdown policies are most effective when they distinguish between different types of activity sites and geographic subregions, when the severity of the lockdown increases with the popularity of the activity site and the population of the subregion, and also when severity of the lockdown increases with the number of infected individuals in the population.

Furthermore, we observe a direct tradeoff between the severity of a lockdown and the resulting number of deceased individuals at the end of the considered time horizon. The proposed lockdown policy synthesis algorithm provides a method to control this tradeoff through the economic impact parameter. By varying the value of this parameter, decision-makers can assess the predicted biological and economic outcomes of an entire suite of lockdown policies, before deploying any of them in practice.

The framework we present is flexible; it can be adapted to incorporate additional modeling considerations and data sources, when such information becomes available. For example, we assume that the economic impact of a lockdown policy is proportional to the demand rate of the corresponding activity sites. However, a more complex economic cost function could easily be incorporated into the optimization objective. Furthermore, additional considerations surrounding the lockdown policy for specific activity sites can be incorporated by adding constraints to the optimization problem. The granularity of the model can also be refined by incorporating additional types of activity sites and a finer partition of the MSA into subregions. Finally, we demonstrate that by including more edges in the multiscale SIRD model, we can significantly reduce the predicted number of deceased individuals at the sole expense of a higher computational cost.

## Supplementary Information

Supplementary Information.
