# Author Correction: Multiscale heterogeneous optimal lockdown control for COVID-19 using geographic information

**DOI:** 10.1038/s41598-022-11546-5

**Published:** 2022-05-05

**Authors:** Cyrus Neary, Murat Cubuktepe, Niklas Lauffer, Xueting Jin, Alexander J. Phillips, Zhe Xu, Daoqin Tong, Ufuk Topcu

**Affiliations:** 1 The University of Texas at Austin, Austin, TX USA; 2 The University of California, Berkeley, Berkeley, CA USA; 3 Arizona State University, Tempe, AZ USA

Correction to: *Scientific Reports* 10.1038/s41598-022-07692-5, published online 10 March 2022

The Supplementary Information published with this Article contained an error.

In the Data Collection and Processing section, under the subheading ‘D.3 Obtaining the Intercity Flow Matrix and Edge Weights’, the following sentence has been removed:

“We show the effects of including more edges in the graph in terms of the obtained results in Table ??.”

The original Supplementary Information 1 file is provided below.

This error has now been corrected in the Supplementary Information file that accompanies the original Article.

## Supplementary Information

Supplementary Information.

