# Supplementary material for: Author Correction: Multiscale heterogeneous optimal lockdown control for COVID-19 using geographic information
Source: Sci Rep. 2022 May 5;12:7371. doi: 10.1038/s41598-022-11546-5 (PMC9070617; doi:10.1038/s41598-022-11546-5)
Supplement: Supplementary file 1 — Supplementary Information. [file 41598_2022_11546_MOESM1_ESM.pdf]

Multiscale Heterogeneous Optimal Lockdown Control for COVID-19 Using Geographic Information: Supplementary Material

Cyrus Neary[1,*], Murat Cubuktepe[1], Niklas Lauffer[1], Xueting Jin[2], Alexander J. Phillips[1], Zhe Xu[2], Daoqin Tong[2], and Ufuk Topcu[1]
[1]The University of Texas at Austin, Austin, TX, USA
[2]Arizona State University, Tempe, AZ, USA
[*]Corresponding author <cneary@utexas.edu>

The supplementary material includes a description of the developed multiscale susceptible, infected, recovered, and deceased (SIRD) model, incorporating lockdown control with this model, details about the formulation of the heterogeneous optimal lockdown problem, data collection, data processing, and the extended results. The necessary data and implementation are included at https://github.com/cyrusneary/multiscaleLockdownCovid19.

A The Multiscale SIRD Model

Each metropolitan statistical area (MSA) is separated into geographically grouped subregions with associated subpopulations. The multiscale SIRD model of a given MSA consists of a directed graph with a node for each subregion. The spread of COVID-19 within the subpopulation of each subregion is governed by a distinct SIRD model. Following the regional model outlined by Della Rossa et al.[1], we model the dynamics of the COVID-19 epidemic spread within the subpopulation of each subregion with the following system of equations.

$$\dot{S}_v(t) = -\beta \sum_{w=1}^{n}\sum_{k=1}^{n} \rho_w(t)\phi_{vw}(t)S_v(t)\frac{\phi_{kw}(t)I_k(t)}{N_w^p}, \forall v \in \{1,\ldots,n\}, \tag{1}$$

$$\dot{I}_v(t) = \beta \sum_{w=1}^{n}\sum_{k=1}^{n} \rho_w(t)\phi_{vw}(t)S_v(t)\frac{\phi_{kw}(t)I_k(t)}{N_w^p} - (\gamma+\Gamma)I_v(t), \forall v \in \{1,\ldots,n\}, \tag{2}$$

$$\dot{D}_v(t) = \Gamma I_v(t), \forall v \in \{1,\ldots,n\}, \tag{3}$$

$$\dot{R}_v(t) = \gamma I_v(t), \forall v \in \{1,\ldots,n\}, \tag{4}$$

$$N_v^p(t) = \sum_{k=1}^{n} \phi_{kv}(t)N_k, \forall v \in \{1,\ldots,n\}, \tag{5}$$

where $S_v(t)$, $I_v(t)$, $D_v(t)$, and $R_v(t)$ are the number of susceptible, infected, recovered, and deceased within subregion v at time t. We use n to denote the number of distinct subregions within the MSA, and we use the symbols v, w, k to index the subregions. We use N_v to represent the population of subregion v, and $N_v^p(t)$ to represent the number of people interacting in subregion v at time t. Furthermore, we use $\phi_{vw}(t)$ to represent the fraction of the population from subregion v visiting subregion w at time t. Finally, we additionally require that $\sum_{w=1}^{n} \phi_{vw}(t) \leq 1$ for all $v, w \in \{1,\ldots,n\}$ and $t \in \{1,\ldots,T\}$.

The parameters in this model are as follows.

- $\beta \in [0,1]$ is a base infection rate of COVID-19, which is assumed to be constant across all subregions.
- $\rho_v \in [0,1]$ is a subregion-specific modifier, that is used to model heterogeneity in the infection rates of different subregions.
- $\gamma \in [0,1]$ is the rate at which infected members of the population recover.

- $\Gamma \in [0,1]$ is the rate at which infected members of the population become deceased.

The summation terms in (1) and (2) capture the spread of COVID-19 between the populations of the various subregions in the MSA. Equation (1) models the rate of change $\dot{S}_v(t)$ of the number of people from subregion v that are susceptible at time t. The value of $\dot{S}_v(t)$ is given by the summation over all subregions w of all the susceptible people from subregion v who become infected while in subregion w. Mathematically, we express this statement as $\dot{S}_v(t) = -\beta \sum_{w=1}^{n} \rho_w(t)\phi_{vw}(t)\frac{S_v(t)\text{NumInfected}_w(t)}{N_w^p(t)}$. Here, $\text{NumInfected}_w(t)$ represents the number of the people in subregion w at time t who are infected, and N_w^p represents the total number of people in subregion w at time t. The value of $\text{NumInfected}_w(t)$ can be approximated as $\sum_{k=1}^{n} \phi_{kw}(t)I_k(t)$. Putting these equations together, we arrive at (1). Meanwhile, (2) uses the same expression to define the rate of change $\dot{I}_v(t)$ of the number of people from subregion v who are infected at time t. However, (2) also includes the term $-(\gamma+\Gamma)I_v(t)$, which captures the decrease in the number of infected people due to recovery (4), or death (3). An estimate for the value of N_w^p is given by (5).

Note that we assume that the members of the population return to their home subregions at the end of each timestep. More specifically, $\phi_{vw}(t)$ represents the fraction of subregion v's population that visits an activity site in subregion w on day t. However, region v's total population N_v stays constant; N_v is *not* reduced by a factor of $\phi_{vw}(t)$ at time $t+1$.

To obtain values for the parameters β, ρ_v, γ, and Γ that have biological significance and that are consistent with our network SIRD model, we use the parameter values that were presented by Della Rossa et al.[1]. The primary focus of their work was to calibrate and analyze a similar network model to ours using data from the start of the pandemic in Italy.

B Adding Activity Sites and Lockdown Control to the Multiscale SIRD Model

In the multiscale SIRD model, the variable driving inter-regional spread is $\phi_{vw}(t)$, which is a dimensionless interaction term between subpopulations. Any attempt at controlling the inter-regional spread of the virus through limitations of population mobility, will thus depend on influencing the values of $\phi_{vw}(t)$.

One possible explanation for people's inter-regional interactions is the frequency at which they visit various types of activity sites within the different subregions. We use m to denote the number of distinct types of activity sides included in the model. For example, *grocery stores*, *restaurants*, and *fitness centers* are all examples of different types of activity sites. We use the symbol i to index of the different types of activity sites. We assume that the interactions at these activity sites are the only factor influencing ϕ_{vw}.

For each subregion $v \in \{1,\dots,n\}$ and activity site type $i \in \{1,\dots,m\}$, we assume there is some fixed *demand rate* d_v^i, which represents the average number of people from subregion v who typically visit an activity site of type i within a day. This quantity is estimated from the SafeGraph data[2].

During the pandemic, many members of the population may either alter their demands for certain types of activity sites or decide to switch to socially distant means of satisfying their needs; for example, switching to online shopping instead of visiting stores. The number of inter-regional interactions will be affected by the fraction of the population choosing to alter their behavior in this way. Let $o_v^i(t)$ denote the number of people from subregion v that have decided not to visit activity sites of type i per day. We assume a linear relationship $o_v^i(t) = A_v^i L_v^i(t) + B_v^i$ holds between $o_v^i(t)$ and the lockdown value $L_v^i(t)$ at any given time t for all subregions v and all activity site types i. Here, A_v^i and B_v^i are parameters to be learned from survey data.

We also assume that each type of activity site (*e.g., grocery stores or restaurants*) within each subregion has some maximum capacity $C_v^i \in \mathbb{N}$ representing the number of people that can be served by activity sites of type i within subregion v. To influence the interactions between subpopulations, and thus the inter-regional spread of the disease, we control the lockdown of each activity site type i within each subregion v by setting the parameter $L_v^i(t)$ which takes values in $[0,1]$ for all times t in the considered time horizon $[0,T]$. This parameter affects the available capacity by scaling the value of C_v^i. That is, $L_v^i(t)C_v^i$ is the total number of people that can be served by an activity site of type i in subregion v at time t.

Furthermore, we introduce the variables $f_{vw}^i(t)$ which represent the average number of people from subregion v who visit activity site i within subregion w at time t. The value of $\phi_{vw}(t)$ — the fraction of subregion v's population who visit *any* type of activity site within subregion w at time t is then given by $\phi_{vw}(t) = \frac{\sum_i f_{vw}^i(t)}{N_v}$.

We model the value of $f_{vw}^i(t)$ at any given time t by comparing the relative values of $d_v^i - o_v^i(t)$ and $C_v^i L_v^i(t)$. If $d_v^i - o_v^i(t) \le C_v^i L_v^i(t)$, the average demand per day for activity site i in subregion v is smaller than the capacity that is being served in that subregion. In this scenario, we have $f_{vv}^i(t) = d_v^i - o_v^i(t)$ and $f_{vw}^i(t) = 0$ for all $w \neq v$; there is no motivation for members of subpopulation v to travel to other subregions in order to visit an activity site of type i. Conversely, if $d_v^i - o_v^i(t) \ge C_v^i L_v^i(t)$, then

we have $f^i_{vv}(t) = C^i_v L^i_v(t)$ and $f^i_{vw}(t) = (d^i_v(t) - o^i_v(t) - C^i_v L^i_v(t))W^i_{vw}$. Here, W^i_{wv} is a value in the range $[0,1]$ that represents the likelihood that a person will choose to travel to subregion w if there is no remaining capacity for activity sites of type i within their home subregion v. These values are estimated from SafeGraph data, as described in §D.3. We may thus concisely write the value of $f^i_{vw}(t)$ for a given time t using the equations $f^i_{vv}(t) = \min(d^i_v - o^i_v(t), C^i_v L^i_v(t))$ and $f^i_{vw}(t) = \max(0, (d^i_v(t) - o^i_v(t) - C^i_v L^i_v(t))W^i_{vw})$ for all $w \neq v$.

We note that while the multiscale SIRD model of (1)-(5) is formulated as continuous-time system of ordinary differential equations, a discretization of the dynamics in time is necessary for numerical simulation. Furthermore, the synthesis of optimal lockdown policies $L^i_v(t)$ in continuous time requires the solution to an infinite-dimensional optimization problem that is computationally intractable due to the aforementioned non-smooth relationship between $L^i_v(t)$ and $\phi_{vw}(t)$. We accordingly use the forward Euler method to obtain a discrete representation of the system dynamics and we model the lockdown policies $L^i_v(t)$ as zero-order-hold signals (i.e. the lockdown factor is assumed to be constant for each discrete timestep). This formulation leads to the discretized form of the system dynamics given below.

Given initial values for the number of susceptible $S_{v,0}$, infected $I_{v,0}$, recovered $R_{v,0}$, and deceased $D_{v,0}$ members of the subpopulations of each subregion v at time $t = 0$, and given a selected lockdown policy $L^i_v(t)$ for each type of activity site within each subregion, we write the *discretized* dynamics of the system as follows.

$$D_v(t+1) = T_s \Gamma I_v(t), \forall t \in \{0, \ldots, T-1\}, \tag{6a}$$

$$I_v(t+1) = T_s\Big(\beta \sum_{w=1}^{n}\sum_{k=1}^{n} \rho_w \phi_{vw}(t) S_v(t) \frac{\phi_{kw}(t) I_k(t)}{N^p_w(t)} - (\gamma + \Gamma) I_v(t)\Big), \forall v \in \{1, ..., n\}, t \in \{0, \ldots, T-1\}, \tag{6b}$$

$$S_v(t+1) = -T_s\Big(\beta \sum_{w=1}^{n}\sum_{k=1}^{n} \rho_w \phi_{vw}(t) S_v(t) \frac{\phi_{kw}(t) I_k(t)}{N^p_w(t)}\Big), \forall v \in \{1, ..., n\}, t \in \{0, \ldots, T-1\}, \tag{6c}$$

$$R_v(t+1) = T_s \gamma I_v(t), \forall v \in \{1, ..., n\}, t \in \{0, \ldots, T-1\} \tag{6d}$$

$$N^p_w(t) = \sum_{v=1}^{n} \phi_{vw}(t) N_v, \forall w \in \{1, ..., n\}, t \in \{0, \ldots, T\}, \tag{6e}$$

$$S_v(0) = S_{v,0}, I_v(0) = I_{v,0}, \forall v \in \{1, ..., n\}, \tag{6f}$$

$$R_v(0) = R_{v,0}, D_v(0) = D_{v,0}, \forall v \in \{1, ..., n\}, \tag{6g}$$

$$o^i_v(t) = A^i_v L^i_v(t) + B^i_v,\ \forall i \in \{1, \ldots, m\}, v \in \{1, \ldots, n\}, t \in \{0, \ldots, T\} \tag{6h}$$

$$f^i_{vv}(t) = \min(d^i_v - o^i_v(t), C^i_v L^i_v(t)), \forall i \in \{1, ..., m\}, v \in \{1, ..., n\}, t \in \{0, ..., T\}, \tag{6i}$$

$$f^i_{vw}(t) = \max(0, (d^i_v - o^i_v(t) - C^i_v L^i_v(t))W^i_{vw}), \forall i \in \{1, \ldots, m\}, v, w \in \{1, \ldots, n\} \text{ s.t. } v \neq w, t \in \{0, \ldots, T\}, \tag{6j}$$

$$\sum_{k=1}^{n} f^i_{kw}(t) \leq C^i_w L^i_w(t), \forall i \in \{1, ..., m\}, w \in \{1, ..., n\}, t \in \{0, \ldots, T\}, \tag{6k}$$

$$\phi_{vw}(t) = \frac{\sum_{i=1}^{m} f^i_{vw}(t)}{N_v}, \forall v \in \{1, ..., n\}, w \in \{1, ..., n\}, t \in \{0, ..., T\}, \tag{6l}$$

$$\sum_{w=1}^{n} \phi_{vw}(t) = \frac{\sum_{i=1}^{m}(d^i_v - o^i_v(t))}{N_v}, \forall v \in \{1, \ldots, n\}, t \in \{0, \ldots, T\}, \tag{6m}$$

where T_s is the sampling period, which is defined to be 1 day in our formulation.

C Formulating the Heterogeneous Optimal Lockdown Problem

In the multiscale SIRD model, the variable driving inter-regional spread is $\phi_{vw}(t)$, the dimensionless interaction term between subpopulations. Given a finite horizon T, our goal is to schedule the partial lockdown capacities of the activity sites within all subregions to minimize the total number of deaths across the entire network at time T.

The constants in this problem are: β, γ, W^i_{vw}, N_v, C^i_v, ρ_v, A^i_v, B^i_v and d^i_v for all $i \in \{1, \ldots, m\}$ and $v, w \in \{1, \ldots, n\}$. The variables in this problem are $S_v(t), I_v(t), R_v(t), D_v(t), N^p_v(t), o^i_v(t), f^i_{vw}(t), \phi_{vw}(t)$, and $L^i_v(t)$ for all $i \in \{1, \ldots, m\}$, for all $v, w \in \{1, \ldots n\}$, and for all $t \in \{0, \ldots, T\}$.

Our objective is

$$\min_{L(t,v), \forall v \in [n], t \in [0,T]} \quad \sum_{v=1}^{n} D_v(T) - \alpha \sum_{v=1}^{n}\sum_{i=1}^{m} d^i_v \sum_{t=1}^{T} L^i_v(t), \tag{7}$$

where $\alpha > 0$ is a positive economic impact parameter. The optimization problem is then defined by the above objective subject to the constraints in (6a)–(6m)

C.1 The Relaxed Optimization Problem

The constraints in (6i) and (6j) involve min and max operators over two affine functions in the variables, respectively. Including these operators in an optimization problem results in a *nonsmooth* problem. Obtaining locally optimal solutions for such nonsmooth problems is significantly more challenging than smooth problems, both in theory and practice. A detailed discussion on the challenges and convergence guarantees for nonsmooth optimization problems can be found in[3]. Therefore, we relax these constraints as follows, i.e., the constraint in (6i) is replaced by

$$\begin{aligned} &f^i_{vv}(t) \leq d^i_v - o^i_v(t), \forall i \in \{1,\dots,m\}, w \in \{1,\dots,n\}, t \in \{0,\dots,T\}, \\ &f^i_{vv}(t) \leq C^i_v L^i_v(t), \forall i \in \{1,\dots,m\}, w \in \{1,\dots,n\}, t \in \{0,\dots,T\}, \end{aligned} \tag{8}$$

and the constraint in (6j) is replaced by

$$\begin{aligned} &f^i_{vw}(t) \geq 0, \forall i \in \{1,\dots,m\}, w \in \{1,\dots,n\}, t \in \{0,\dots,T\}, \\ &f^i_{vw}(t) \geq (d^i_v - o^i_v(t) - C^i_v L^i_v(t)) W^i_{vw}, \forall i \in \{1,\dots,m\}, v \in \{1,\dots,n\} \,\text{s.t.}\, v \neq w, t \in \{0,\dots,T\}. \end{aligned} \tag{9}$$

The practical implication of these modifications is, given a subregion v and an activity site of type i, we allow more people to travel to other cities to fulfill their demand by relaxing the constraint in (6j). This relaxation may increase the inter-regional spread of the disease. On the other hand, relaxing the constraints in (6i) may result in fewer people staying in their subregion while fulfilling their demand, which may decrease the regional spread of the disease.

C.2 Computing Locally Optimal Solutions to the Optimization Problem

The optimization problem in (6a)–(6m) with the objective (7) is nonconvex as the functions in the constraints (6b)–(6c) are nonlinear in the problem variables ϕ, S, and I. Therefore, it is in general computationally intractable to compute a globally optimal solution to the nonconvex optimization problem[4]. We thus focus on computing a locally optimal solution to the nonconvex problem in a reasonable time.

Our method is based on sequential convex programming (SCP) approaches[5,6]. SCP methods iteratively compute a locally optimal solution to a nonconvex problem by linearizing the nonconvex constraints around a previous solution. However, when a feasible nonconvex problem is linearized around a previous solution, it may result in an infeasible convex subproblem[5]. To ensure the feasability of the linearized problem, we add *slack variables* to the linearized constraints. Additionally, the linearization may not be accurate if the solutions to the linearized problem deviate significantly from the previous solution. Therefore, we utilize trust region constraints[5,7,8] to ensure that the linearized problems accurately approximate the nonconvex optimization problem.

Linearizing the nonconvex constraints. For a given v, w, k, and t, the function in the constraints (6b)–(6c)

$$h_{vwk}(t) := \rho_w \phi_{vw}(t) S_v(t) \frac{\phi_{kw}(t) I_k(t)}{N^p_w(t)}$$

are nonlinear in the following problem variables $x := [\phi_{vw}(t), \phi_{kw}(t), S_v(t), I_k(t)]$. In our optimization approach, we fix the term $N^p_w(t)$ in each iteration, which improves the efficiency and numerical stability. Therefore, we linearize the function $h_{vwk}(t)$ around a previous solution $\hat{x} = [\hat{\phi}_{vw}(t), \hat{\phi}_{kw}(t), \hat{S}_v(t), \hat{I}_k(t)]$ as

$$\hat{h}_{vwk}(t) := h_{vwk}(t;\hat{x}) + \nabla_{\hat{x}} h_{vwk}(t)^\top (x - \hat{x}),$$

where $h_{vwk}(t;\hat{x})$ denotes the evaluation of the function $h_{vwk}(t)$ at $\hat{x}$ and $\nabla_{\hat{x}} h_{vwk}(t)$ denotes the gradient of the function $h_{vwk}(t)$ at $\hat{x}$. Therefore, we linearize the constraints (6b)–(6c) as

$$I_v(t+1) = T_s\Big(\beta \sum_{w=1}^{n} \sum_{k=1}^{n} \hat{h}_{vwk}(t) - \gamma I_v(t)\Big), \forall v \in \{1,\dots,n\}, t \in \{0,\dots,T-1\}, \tag{10}$$

$$S_v(t+1) = -T_s\Big(\beta \sum_{w=1}^{n} \sum_{k=1}^{n} \hat{h}_{vwk}(t)\Big), \forall v \in \{1,\dots,n\}, t \in \{0,\dots,T-1\} \tag{11}$$

which are convex in the problem variables.

Adding slack variables and trust region constraints. Recall that the affine constraints in (10)–(11) may be infeasible, or the linearization may be inaccurate if the solution deviates significantly from the previous computed solution. We alleviate these drawbacks by adding *slack variables* $k_{vwk}(t)$ and $l_{vwk}(t) \in \mathbb{R}$ to (10) and (11) respectively, and trust region constraints to the ϕ variables as

$$I_v(t+1) + k_{vwk}(t) = T_s\Big(\beta \sum_{w=1}^{n}\sum_{k=1}^{n} \hat{h}_{vwk}(t) - \gamma I_v(t)\Big), \forall v \in \{1,\dots,n\}, t \in \{0,\dots,T-1\}, \tag{12}$$

$$S_v(t+1) + l_{vwk}(t) = -T_s\Big(\beta \sum_{w=1}^{n}\sum_{k=1}^{n} \hat{h}_{vwk}(t)\Big), \forall v \in \{1,\dots,n\}, t \in \{0,\dots,T-1\}, \tag{13}$$

$$|\hat{\phi}_{vw}(t) - \phi_{vw}(t)| \le \sigma, \forall v \in \{1,\dots,n\}, w \in \{1,\dots,n\}, t \in \{0,\dots,T-1\}, \tag{14}$$

where σ is the size of the trust region to restrict the set of allowed policies in the linearized problem. Finally, we augment the cost function in (7) with the term $\lambda \sum_{t=1}^{T-1}\sum_{v=1}^{n}\sum_{w=1}^{n}\sum_{k=1}^{n} |k_{vwk}(t)| + |l_{vwk}(t)|$ to ensure that we minimize the violation of the constraints, where λ is a large enough positive constant to minimize the violations in the linearized constraints.

Checking the validity of the solution and updating the trust region. After solving the relaxed problem and obtaining solutions for the interaction terms $\phi_{vw}(t)$ for all v, w, and t, we compute the *actual* cost of the solution by implementing the solution for the interaction terms and running the multiscale SIRD dynamics, defined in (6a)–(6g). If the actual cost for the obtained solution for the computed interaction terms is lower than the best-obtained cost, we update our solution and linearize the nonconvex optimization problem around the updated solution. When we update the solution, we also increase the size σ of the trust region. On the other hand, if the actual cost is larger, we conclude that our approximation is not accurate and shrink the trust region without updating the solution, and re-solve the linearized optimization problem. If the size σ of the trust region is sufficiently small (indicating that our approximation to the nonconvex optimization problem is accurate) and the algorithm cannot compute a solution that obtains a lower objective value than the last obtained solution, we conclude that the last obtained solution is locally optimal.

D Data Collection and Processing

D.1 Data Collection

Our study was conducted on six of the largest metropolitan statistical areas (MSAs) in the United States, including New York, Los Angeles, Chicago, Dallas-Fort Worth, Phoenix, and Seattle. These areas are selected considering that they are among the fifteen most populated MSAs and are distributed in different parts of the country. In each MSA, the foot traffic information of activity sites in April 2020 are collected from SafeGraph[2]. The data include information on activity site ID, activity site location, NAICS code describing the particular type of business activity associated with each activity site, the census block groups with at least four residents who visited the activity site, and the associated amount of visitors. Based on the NAICS codes, we extract six main types of activity sites, including grocery stores, restaurants, hotels, pharmacies, fitness centers, and physicians.

D.2 Data Processing

As the SafeGraph data only includes the foot traffic information pertaining to a subset of the total population, we first calibrate the data by scaling all counts by the number of devices $SafeGraphDevices_v$ used to conduct the data collection for each subregion v. Using the calibrated foot traffic data for each type of activity site, we calculate the demand rate d_v^i for each subregion v and activity site type i. We recall that d_v^i is the average number of people from subregion v visiting an activity site of type i per day. We compute the value of d_v^i by

$$d_v^i = \frac{Population_v}{SafeGraphDevices_v} \times \sum_{w=1}^{n} freq^i \times FootTraffic_{vw}^i.$$

Here, $freq^i$ denotes the frequency at which a person typically visits an activity site of type i per month. This scaling is necessary as the SafeGraph dataset only reports the number of unique visitors to an activity site per month; even if a person visits an activity site type multiple times in a month, SafeGraph only reports a single visit. Meanwhile, $Population_v$ represents the total population in subregion v, and $FootTraffic_{vw}^i$ is the number of people in the SafeGraph dataset from subregion v visiting an activity site of type i in subregion w in a month.

Additionally, using data showing COVID-19 cases per zip code or per county on specific dates[9–14], we find the number of cases in each subregion on a specific date that can be used as an initial condition in (6f) and (6g).

D.3 Obtaining the Intercity Flow Matrix and Edge Weights

We also compute parameters W^i_{vw} using SafeGraph data. We recall that W^i_{vw} are included in (6j) and represent the likelihood a person from subregion v would visit an activity site of type i in subregion w, given there is not enough capacity for activity sites of type i in subregion v. We compute the values of parameters W^i_{vw} by

$$W^i_{vw} = \frac{FootTraffic^i_{vw}}{\sum_{w' \in Adj_k(v)} FootTraffic^i_{vw'}}. \tag{15}$$

Here, $Adj_k(v)$ is the set of subregions w for which there exists a directed edge from subregion v to w. For any value k, representing the maximum number of outgoing edges from a subregion, we compute $Adj_k(v)$ using the values of $FootTraffic^i_{vw}$. Specifically, we compute

$$TotalTraffic_{vw} = \sum_{i=1}^{m} FootTraffic^i_{vw}, \tag{16}$$

and we define $Adj_k(v)$ as the set containing the subregions w with the k largest values of $TotalTraffic_{vw}$. We show the effects of including more edges in the graph in terms of the obtained results in Table **??**.

E Extended Results

In this section, we demonstrate the effects of the presented multiscale heterogeneous lockdown policies on the dynamics of the multiscale SIRD model that are estimated using data for Phoenix, Seattle, New York, Chicago, Los Angeles, and Dallas-Fort Worth MSAs. In the following figures, we visualize the resulting dynamics without any imposed lockdowns in the left column, and with an optimal lockdown in the right column for each MSA. We note that in all MSAs, there is no noticeable peak for the infections after imposing the lockdown policy, and the number of cumulative infections and deceased people decreases significantly.

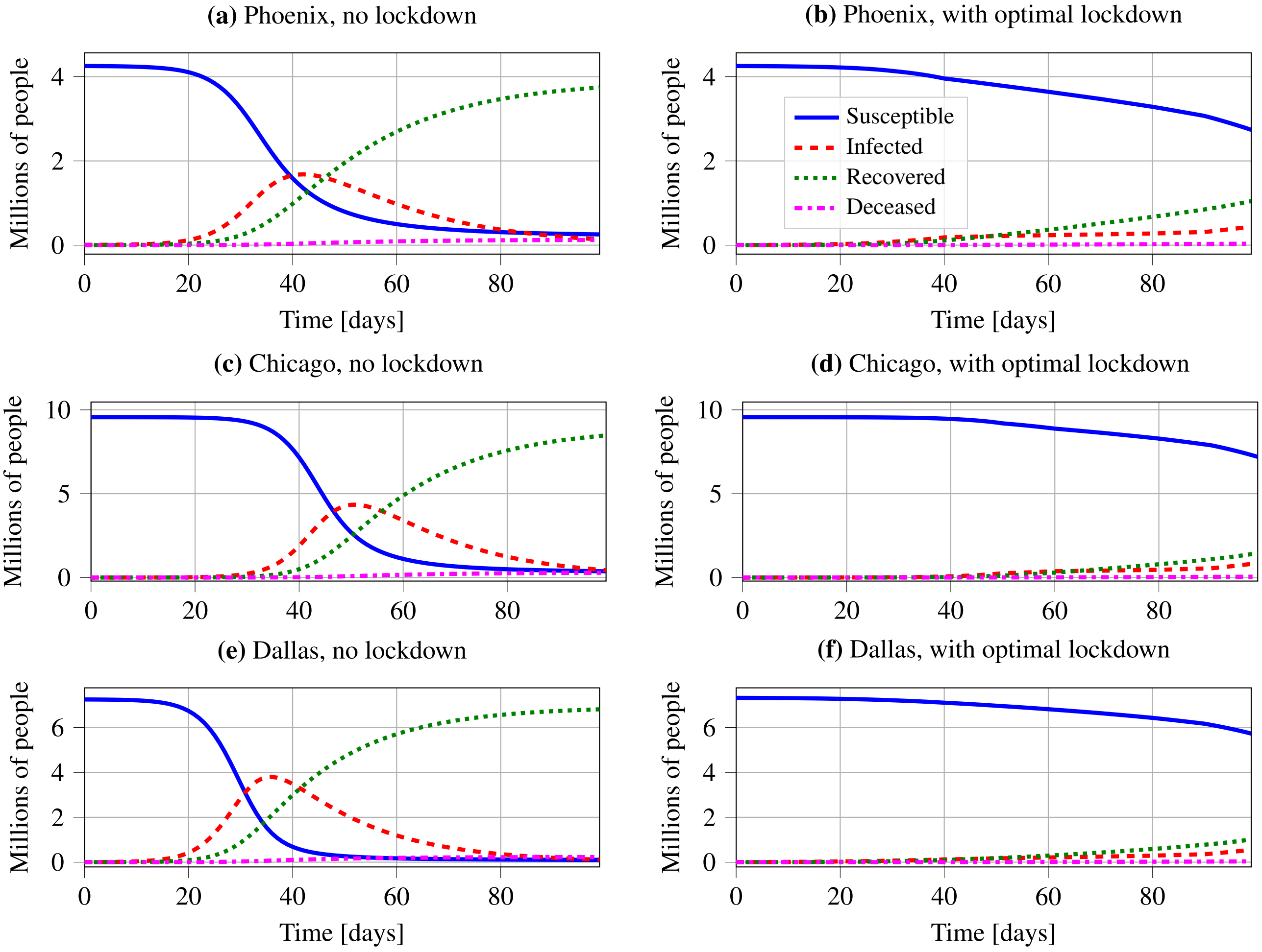
(a) Phoenix, no lockdown
(b) Phoenix, with optimal lockdown
(c) Chicago, no lockdown
(d) Chicago, with optimal lockdown
(e) Dallas, no lockdown
(f) Dallas, with optimal lockdown
Millions of people
Time [days]
Susceptible
Infected
Recovered
Deceased

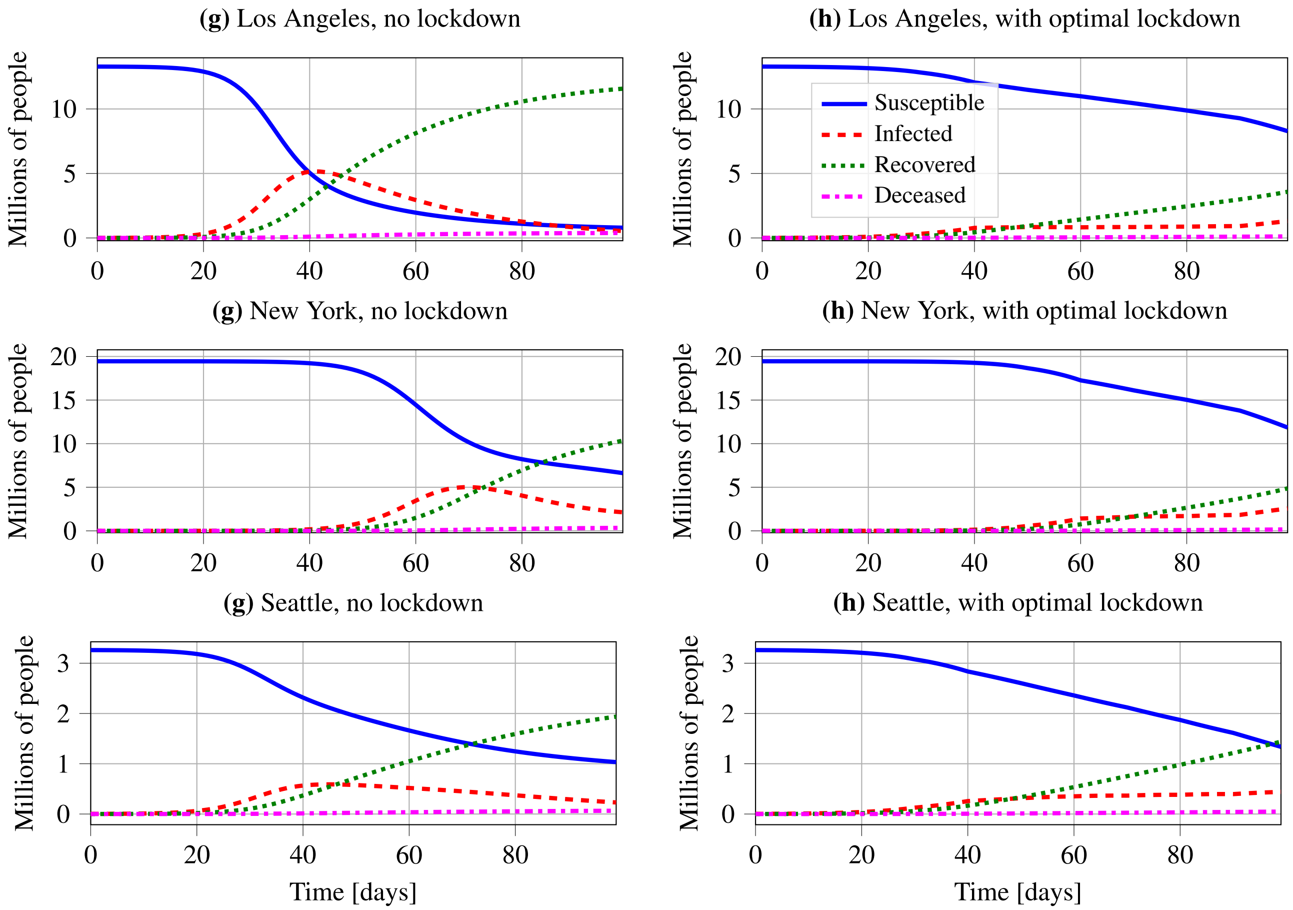

Figure 1. Results from all considered MSAs. In the left column, we show the dynamics of the multiscale SIRD model without any lockdown policy. In the right column, we show the dynamics after applying an optimal lockdown policy. In all cases, we observe a significant decrease both in the peak infection rate and also in the number of cumulative infected and deceased individuals.

References

1. Della Rossa, F. *et al.* A Network Model of Italy Shows that Intermittent Regional Strategies can Alleviate the COVID-19 Epidemic. *Nat. communications* **11**, 5106, DOI: https://doi.org/10.1038/s41467-020-18827-5 (2020).
2. SafeGraph: U.S. Consumer Activity During COVID-19 Pandemic. https://www.safegraph.com/data-examples/covid19-commerce-patterns. Accessed: 2021-05-20.
3. Kungurtsev, V., Mitchell, T. & Vyhlidal, T. A Comparison of Nonsmooth, Nonconvex, Constrained Optimization Solvers for the Design of Time-Delay Compensators. *arXiv preprint arXiv:1812.11630* (2018).
4. Lobo, M. S., Vandenberghe, L., Boyd, S. & Lebret, H. Applications of Second-Order Cone Programming. *Linear algebra its applications* **284**, 193–228, DOI: https://doi.org/10.1016/S0024-3795(98)10032-0 (1998).
5. Mao, Y., Szmuk, M., Xu, X. & Açikmese, B. Successive Convexification: A Superlinearly Convergent Algorithm for Non-convex Optimal Control Problems. *arXiv preprint arXiv:1804.06539* (2018).
6. Bolte, J. & Pauwels, E. Majorization-Minimization Procedures and Convergence of SQP Methods for Semi-algebraic and Tame Programs. *Math. Oper. Res.* **41**, 442–465, DOI: https://doi.org/10.1287/moor.2015.0735 (2016).
7. Chen, X., Niu, L. & Yuan, Y. Optimality Conditions and a Smoothing Trust Region Newton Method for Nonlipschitz Optimization. *SIAM J. on Optim.* **23**, 1528–1552, DOI: https://doi.org/10.1137/120871390 (2013).
8. Yuan, Y.-x. Recent Advances in Trust Region Algorithms. *Math. Program.* **151**, 249–281, DOI: https://doi.org/10.1007/s10107-015-0893-2 (2015).
9. COVID-19 Cases by Zip Code. https://data.tempe.gov/datasets/tempegov::covid-19-cases-by-zip-code/about. Accessed on 2021-07-04.
10. Daily COVID-19 Outbreak Summary. https://kingcounty.gov/depts/health/covid-19/data/daily-summary.aspx. Accessed on 2021-07-04.
11. COVID-19 Time-Series Metrics by County and State - Statewide COVID-19 Cases Deaths Tests. https://data.chhs.ca.gov/dataset/covid-19-time-series-metrics-by-county-and-state/resource/046cdd2b-31e5-4d34-9ed3-b48cdbc4be7a. Accessed on 2021-07-04.
12. COVID-19 County Cases, Tests, and Deaths by Day. https://www.dph.illinois.gov/content/covid-19-county-cases-tests-and-deaths-day. Accessed on 2021-07-04.
13. Dallas County COVID-19 Summaries. https://www.dallascounty.org/departments/dchhs/2019-novel-coronavirus/summaries.php. Accessed on 2021-07-04.
14. New York State Department of Health COVID-19 Tracker. https://covid19tracker.health.ny.gov/views/NYS-COVID19-Tracker/NYSDOHCOVID-19Tracker-Map. Accessed on 2021-07-04.
